# Construction of Raman spectroscopic fingerprints for the detection of Fusarium wilt of banana in Taiwan

**DOI:** 10.1371/journal.pone.0230330

**Published:** 2020-03-16

**Authors:** Yi-Jia Lin, Hsuan-Kai Lin, Ying-Hong Lin

**Affiliations:** 1 Department of Plant Medicine, National Pingtung University of Science and Technology, Pingtung, Taiwan; 2 Institute of Materials Engineering, National Pingtung University of Science and Technology, Pingtung, Taiwan; National Cheng Kung University, TAIWAN

## Abstract

Banana (*Musa* sp.) is cultivated worldwide and is one of the most popular fruits. The soil-borne fungal disease Fusarium wilt of banana (FWB), commonly known as Panama disease, is caused by *Fusarium oxysporum* f. sp. *cubense* (Foc) and is a highly lethal vascular fungal disease in banana plants. Raman spectroscopy, an emerging laser-based technology based on Raman scattering, has been used for the qualitative characterization of biological tissues such as foodborne pathogens, cancer cells, and melamine. In this study, we describe a Raman spectroscopic technique that could potentially be used as a method for diagnosing FWB. To that end, the Raman fingerprints of Foc (including mycelia and conidia) and Foc-infected banana pseudostems with varying levels of symptoms were determined. Our results showed that eight, eleven, and eleven characteristic surface-enhanced Raman spectroscopy peaks were observed in the mycelia, microconidia, and macroconidia of Foc, respectively. In addition, we constructed the Raman spectroscopic fingerprints of banana pseudostem samples with varying levels of symptoms in order to be able to differentiate Foc-infected bananas from healthy bananas. The rate at which FWB was detected in asymptomatic Foc-infected samples by using the spectral method was 76.2%, which was comparable to the rates previously reported for other FWB detection methods based on real-time PCR assays, suggesting that the spectral method described herein could potentially serve as an alternative tool for detecting FWB in fields. As such, we hope that the developed spectral method will open up new possibilities for the on-site diagnosis of FWB.

## Introduction

Fusarium wilt of banana (FWB), which is commonly known as Panama disease and is caused by the soil-borne fungal pathogen *Fusarium oxysporum* f. sp. *cubense* (Foc), is a highly lethal vascular fungal disease in banana plants, and is thus reported to be one of the major limiting factors for banana production worldwide [1].

When cultured solid media, e.g. potato dextrose agar (PDA), Foc can grow aerial mycelia that are white in color and produce two types of asexual spores: microconidia and macroconidia. Microconidia are numerous, elliptical to allantoid in shape, and one- or two-celled. Macroconidia are sickle shaped and three- to five-celled [2]. Mycelia and both types of spores mentioned above are commonly found on infected banana plants [3]. The mycelia can penetrate roots and then move through the root cortex, finally reaching the xylem vessels. The pathogen remains in the vessels and travels through them. The microconidia germinate when their upward movement is interrupted, after which the germinated hyphae will penetrate the upper wall of vessels and more microconidia will be produced in the neighboring vessels. Finally, the vascular tissues of an infected banana plant are filled with pathogen structures, leading to the disruption of water translocation within the plant and causing yellowing and wilting of the older leaves that then progresses to the younger leaves until the whole plant collapses [3].

In order to reduce the impact of FWB outbreaks, the development of a reliable method for on-site detection of Foc is important, as efficient methods for diagnosing crop diseases are greatly beneficial to the improvement of disease management strategies. The conventional methods used to diagnose FWB mainly rely on the observable symptoms of samples and the isolation, culturing, and morphological observations of the pathogen itself, as well as on further pathogenicity tests for confirmatory diagnosis [1]. Traditional pathogen isolation and further pathogenicity tests of Foc, however, require a few months, a length of time which can sometimes allow Foc to spread dramatically and cause severe epidemics [4]. In addition, diagnosing FWB with such methods is sometimes difficult, particularly when the symptoms are not typical and clear, a situation which could cause the diagnostician to confuse it with other foliar fungal diseases (e.g. Anthracnose, Deightoniella leaf spot Alternaria speckle, Cordana leaf spot).

To date, a variety of advanced molecular techniques are available for the identification and detection of Foc, such as polymerase chain reaction (PCR) [5, 6], multiplex PCR [7], real time-PCR [8, 9], loop-mediated isothermal amplification (LAMP) [10], real-time LAMP (RealAmp) [11, 12], and insulated isothermal PCR (iiPCR) assays [1]. These molecular methods are more reliable and faster than the conventional methods and can serve as efficacious tools to monitor the Foc population in banana tissues or soil. However, these established detection methods still require some time for DNA extraction. Moreover, high-quality DNA are not easily extracted from some materials, such as Foc chlamydospores and Foc-contaminated soil, which limits the applicability of these molecular methods.

Raman spectroscopy is a nondestructive, emerging laser-based analytical technique based on Raman scattering that has been shown to be a useful tool for the rapid detection and quantification of biotic and abiotic samples such as foodborne pathogens [13, 14, 15], cancers [16, 17], harmful chemical residues (i.e., synthetic chemical pesticides) [18, 19], and melamine [20, 21], with surface-enhanced Raman spectroscopy (SERS) being particularly useful in this regard. The vibrational Raman spectra of biological specimens can reflect their specific overall molecular compositions [13, 17], including proteins, nucleic acids, lipids, carbohydrates, and inorganic crystals [22], which is useful for species identification [22, 23].

However, the scattered light intensity of Raman scattering is typically extremely weak [17], and auto-fluorescence is often present in biological samples, making the detection of Raman signals difficult [24]. These disadvantages make it challenging to use conventional Raman spectroscopy for practical applications involving biological detection.

The limitations of conventional Raman spectroscopy may be overcome by SERS, which can enhance Raman signal intensity by exploiting the surface interactions between a target and a nanoscale metal (such as silver or gold) [25]. SERS offers unique advantages such as high sensitivity, simple treatment, and low scanning time, and has been widely used for various practical applications [26, 27]. SERS, with its high sensitivity, little-to-no sample processing, and rapid detection times, was shown to be a potentially useful tool in clinical medicine for the earlier diagnosis of neurological, cardiovascular, and viral diseases, as well as cancers [28]. To the best of our knowledge, however, there have been no reports regarding the use of Raman spectroscopy or SERS for the detection of FWB. The purpose of this study, therefore, was to construct specialized Raman spectroscopic fingerprints for further detection of FWB based on SERS. In the study, we successfully determined Raman fingerprints related to the macroconidia, microconidia, and mycelia of Foc. We also used a field-detection assay to evaluate the feasibility of the SERS method. Here, we present the results of the first prospective vibrational spectroscopic study in which FWB could be detected without DNA extraction by using the SERS method.

## Materials and methods

### Pathogens and growth conditions

Foc isolates and other fungal pathogens collected from banana including *Colletotrichum musae* (Colm, which causes Anthracnose of banana), *Deightoniella torulosa* (Dt, which causes Deightoniella leaf spot), *Alternaria alternata* (Aa, which causes Alternaria speckle of banana), *Botryosphaeria dothidea* (Bd, which causes Crown rot of banana), and *Cordana musae* (CorM, which causes Cordana leaf spot of banana) were used in this study. A single spore culture of each tested Foc and fungal isolate was grown on a potato dextrose agar (PDA) plate (200 g/l of potato extracts, 1% glucose, and 2% agar).

### Sample preparation for SERS measurements

Fresh fungal mycelia (100 mg) and banana pseudostems (1 g) were homogenized using a taco^™^Prep Bead Beater (GeneReach Biotechnology Corp., Taichung, Taiwan) according to the manufacturer's instructions. The number of conidia was counted with a glass haemacytometer under a microscope (Carl Zeiss, Axioskop 2 plus, Germany). Aliquots of the homogenized fungal mycelia, banana pseudostems, and quantified conidia were loaded onto a Raman SERS-chip (Labguide Co., Ltd., Taipei, Taiwan) illuminated with laser light and detected by a QE65 Pro spectrometer (Ocean optics, Inc., Dunedin, USA) for SERS measurements.

### SERS measurements

A portable QE65 Pro Raman Spectrometer System (Ocean optics, Inc., Dunedin, USA) equipped with a 785-nm near-infrared diode laser source was used in this study. The samples of fungal mycelia, fungal spores, and banana pseudostems were detected with the aforementioned QE65 Pro Raman spectrometer at a laser power of 20 mW, and the integration time for the three types of samples were 15, 10, and 3 s, respectively. The strong [29] and reproducible [30] Raman peaks among the measured SERS spectra were selected as the fingerprinting/characteristics of the Raman spectra.

### Sampling criteria of symptomatic banana samples for SERS measurements and molecular detections

Foc-infected banana pseudostems with varying symptoms were used for SERS measurement and molecular detection assays. The sampling criteria used for these infected banana pseudostems were previously described in detail elsewhere [1]. Specifically, we collected a total of 73 banana pseudostem samples (among them, there were 21 asymptomatic pseudostems and 52 symptomatic pseudostems, including 22 mildly symptomatic, 11 moderately symptomatic, and 19 severely symptomatic pseudostems) and 15 samples of banana infected with fungal pathogens other than Foc (3 samples for each disease) from 12 different fields that had been strongly affected by FWB. Necrosis covering less than 1/3 of the total area of a pseudostem, less than 2/3 but equal to or more than 1/3 of the total area, and equal to or more than 2/3 of the total area were classified as mild, moderate, and severe symptoms, respectively. The field-infected banana pseudostems showing varying symptoms were washed, surface-sterilized with 1% sodium hypochlorite (NaHClO), rinsed in sterile water, and dried under a laminar flow hood to eliminate epiphytic microbes. The surface-sterilized banana pseudostems were then cut into 1 cm^2^ sections and put onto a Nash PCNB agar medium (1.5% peptone, 2% agar, 0.1% KH_2_PO_4_, 0.05% MgSO_4_^.^7H_2_O, 0.1% pentachloronitrobenzene, 0.03% streptomycin, and 0.1% neomycin) for a plate-out assay [31]. Simultaneously, a piece of the banana pseudostems surrounding each section was used for further SERS measurements and molecular detections. In addition, the 21 asymptomatic pseudostems were also incubated at 28°C for 2 months in a growth chamber to make sure the samples were Foc-infected.

### Molecular detections of FWB

Three previously published molecular detection methods, namely MDIP (molecular detection of isolated pathogen) [5], IPDP (*in*-*planta* detection with PCR) [4], and IPDQP (*in*-*planta* detection with real-time PCR) [8], were also performed in this study in order to compare their results with the SERS measurements. The pathogen isolation and DNA extraction for the MDIP were previously described in detail elsewhere [5]. Specifically, the surface-sterilized field-infected banana pseudostems showing varying symptoms were used for the plate-out assay. After the plate-out assay, the pathogens grown on the Nash-PCNB agar medium were used for DNA extraction as previously described in detail elsewhere [5]. The DNA samples (50 ng) of the pathogens were then used for further PCR identification (specifically, using MDIP) according to the procedures described in Lin *et al*. [5]. In addition, a piece of each of the banana pseudostems (0.3 g) showing varying symptoms was directly used for DNA extraction and further PCR (specifically, using IPDP) or real-time PCR (specifically, using IPDQP) assays. The further procedures of PCR for IPDP and real-time PCR for IPDQP were performed as previously described in [5] and [8], respectively.

## Results and discussion

To evaluate the potential of the Raman spectroscopy assay for the rapid differentiation of banana pathogens, the SERS spectra databases of the fungal pathogens on banana in Taiwan were built, including those for Foc, Colm (which causes Anthracnose of banana), Dt (which causes Deightoniella leaf spot), Aa (which causes Alternaria speckle of banana), Bd (which causes Botryosphaeria crown rot of banana), and Corm (which causes Cordana leaf spot of banana).

**Fig 1:**
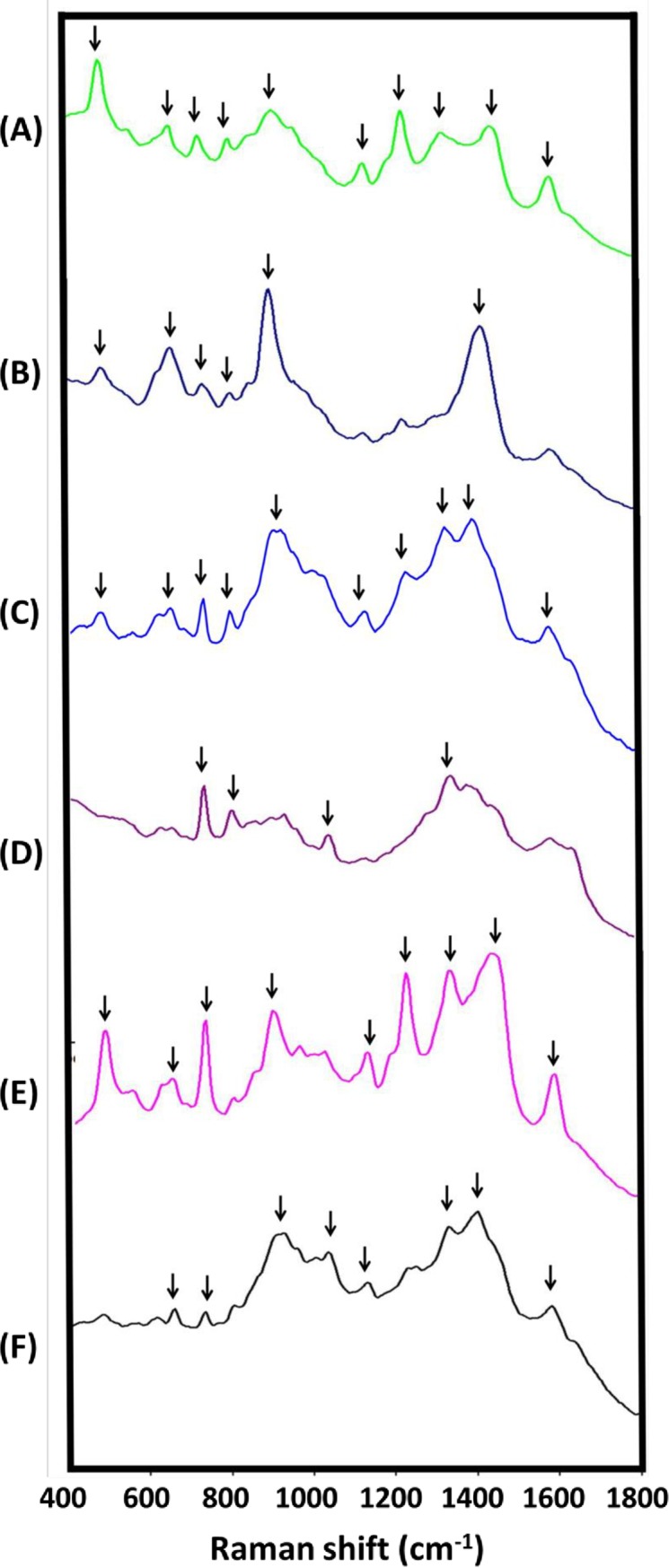
Raman spectra fingerprints of mycelia of the fungal pathogens collected from banana. The Raman spectra databases of the fungal pathogens on banana in Taiwan were built, including databases for (A) *Botryosphaeria dothidea* (which causes Crown rot of banana, YJC-F004), (B) *Deightoniella torulosa* (which causes Deightoniella leaf spot, PM-YJL-F119), (C) *Alternaria alternata* (which causes Alternaria speckle of banana, PM-TYC-F003), (D) *Cordana musae* (which causes Cordana leaf spot of banana, LNH-F001), (E) *Colletotrichum musae* (which causes Anthracnose of banana, PM-YHL-F001), and (F) *Fusarium oxysporum* f. sp. *cubense* tropical race 4 (which causes Fusarium wilt of banana, PM-TYC-F040). Average Raman spectra were obtained from five independent replications of the surface-enhanced Raman spectroscopy measurements.

**Fig 2:**
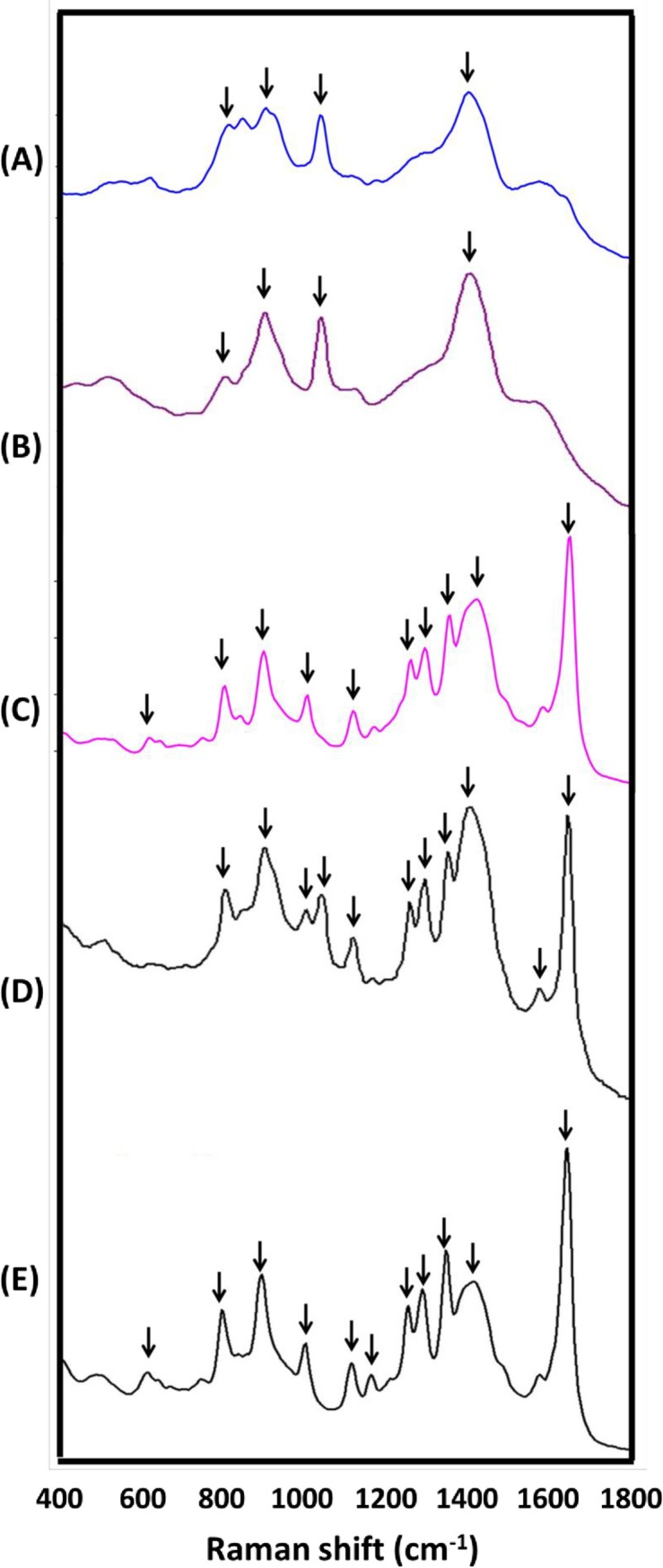
Raman fingerprints of microconidia (A to D) and macroconidia (E) of the fungal pathogens collected from banana. The Raman spectra databases of the fungal pathogens on banana in Taiwan were built, including databases for (A) *Alternaria alternata* (which causes Alternaria speckle of banana, PM-TYC-F003), (B) *Cordana musae* (which causes Cordana leaf spot of banana, LNH-F001), (C) *Colletotrichum musae* (which causes Anthracnose of banana, PM-YHL-F001), and (D & E) *Fusarium oxysporum* f. sp. *cubense* tropical race 4 (which causes Fusarium wilt of banana, PM-TYC-F040). Average Raman spectra were obtained from five independent replications of the surface-enhanced Raman spectroscopy measurements.

The mean SERS spectra of the tested mycelia and conidia covering the spectral range of 400–1800 nm are presented in Fig 1 and Fig 2, respectively. The characteristic band assignments of the SERS spectra are listed in Table 1 (mycelia) and Table 2 (conidia). As shown, eight (652, 733, 896, 1063, 1163, 1332, 1416, and 1590 cm^−1^; Fig 1F), eleven (802, 896, 1003, 1039, 1113, 1255, 1289, 1346, 1416, 1576, and 1639 cm^−1^; Fig 2D), and eleven (614, 802, 896, 1003, 1113, 1189, 1255, 1289, 1346, 1416, and 1639 cm^−1^; Fig 2E) characteristic SERS peaks were observed in the mycelia, microconidia, and macroconidia of Foc, respectively.

**Table 1:** Raman shift and putative peak assignments of the SERS spectra from mycelia of the common causative pathogens used in this study.

Raman shift (cm^-1^)	Pathogens/Diseases						Peak assignment	Ref.
	*Botryosphaeria dothidea/*Botryosphaeria crown rot	*Deightoniella torulosa*/Deightoniella leaf spot	*Alternaria alternata*/Alternaria speckle	*Cordana musae*/Cordana leaf spot	*Colletotrichum musae*/Anthracnose disease	*Fusarium oxysporum* f. sp. *cubense tropical* race 4/Fusarium wilt	Assignment	
483	+						Trilinolenin	[41]
487		+	+		+		δ(C-C = O) deformations	[42]
628	+						Adenine	[43]
632		+	+				2-deoxy-D-erythro-pentose	[43]
652					+	+	Guanine	[32]
726	+						Triolein or trilinolein	[41]
733		+	+	+	+	+	Trehalose	[41]
802	+	+	+	+			C-C ring breathing of fatty acid	[32, 33]
896	+	+	+		+	+	CH_2_ groups of fatty acid	[34]
1063				+		+	C-C stretching of lecithin	[41]
1163	+		+		+	+	Adenine	[41]
1212	+		+		+		Cytosine	[45]
1332	+		+	+	+	+	Dextrose or β-d-glucose	[41]
1416		+	+			+	Chitin	[41]
1440	+				+		Triolein or trilinolein	[41]
1590	+		+		+	+	Unassigned	None

**Table 2:** Raman shift and putative peak assignments of the SERS spectra from conidia of the common causative pathogens used in this study.

Raman shift (cm^-1^)	Pathogens/Diseases/Spores					Peak assignment	Ref.
	*Alternaria alternata*/Alternaria speckle/Microconidia	*Cordana musae*/Cordana leaf spot/Microconidia	*Colletotrichum musae*/Anthracnose disease/Microconidia	*Fusarium oxysporum* f. sp. *cubense* tropical race 4 (Foc race 4)/Fusarium wilt/Microconidia	*Foc* race 4/Fusarium wilt/Macroconidia	Assignment	
614			+		+	Amylopectin or cellulose	[41]
802	+	+	+	+	+	C-C ring breathing of fatty acid	[32, 33]
896	+	+	+	+	+	CH_2_ groups of fatty acid	[34]
1003			+	+	+	C-C aromatic ring of phenylalanine	[32]
1039	+	+		+		Pyridine breathing vibrations	[40]
1113			+	+	+	Deformational vibration of tryptophan	[32]
1189					+	In plane deformation vibrations of C-N	[44]
1255			+	+	+	Amide III of collagen	[30]
1289			+	+	+	C-C stretching of unbranched saturated fatty acids	[34]
1346			+	+	+	CH_2_ of tryptophan	[32]
1416	+	+	+	+	+	Chitin	[41]
1576				+		Nucleic acid	[35, 36]
1639			+	+	+	C-C stretching ring or in-plane C-H bending	[37]

It is notable that the 652 cm^−1^ peak, which corresponds to guanine [32], was only present in the mycelia of Foc (Table 1). The Raman spectra reflected from these characteristic SERS peaks may be potentially useful for the identification of Foc. Several characteristic peaks were observed from most of the tested pathogens. Specifically, most of the tested conidia featured peaks around 802, 896, and 1416 cm^-1^ (Table 2), while most of the tested mycelia also featured SERS peaks around 733 (except for Bd), 896 (except for Corm), and 1332 (except for Dt) (Table 1) cm^-1^. The peaks around 802 and 896 cm^−1^ may have been from C-C ring breathing [32, 33] and CH_2_ groups of fatty acid chain [34], respectively. Meanwhile, the major assignments of the peaks around 733, 1332, and 1416 cm^−1^ were to saccharides (trehalose, dextrose, and chitin, respectively). The vibration mode and major assignment of the peaks around 1590 cm^−1^, however, were not yet determined.

Comparisons of the SERS patterns showed high similarity between the conidia of Foc (Fig 2D & 2E) and Colm (Fig 2C). This was especially true for the peaks around 802, 896, 1003, 1113, 1255, 1289, 1346, 1416, and 1639 cm^−1^, as they were observed in all of the conidia samples (that is, both macroconidia and microconidia) of Foc and Colm (Table 2). However, the SERS peak at 1189 cm^-1^ attributed to in-plane deformation vibrations of C-N was only observed in the macroconidia of Foc, while the peaks around 1576 cm^-1^, which may correspond to nucleic acid [35, 36], were only observed in the microconidia of Foc. The spectral region at 1003 and 1113 cm^−1^ can mainly be attributed to the C-C aromatic ring of phenylalanine and deformational vibration of tryptophan [32], respectively, and the bands at 1255 and 1289 cm^−1^ may be attributable to the amide III of collagen [30] and C-C stretching of unbranched saturated fatty acids [34], respectively. The peaks of 1346 and 1639 cm^−1^ were possibly related to the CH_2_ of tryptophan [32] and C-C stretching ring or in-plane C-H bending, respectively [37]. In addition, we tested Bd (YJC-F004) and Dt (PM-YJL-F119) on potato dextrose agar, yeast peptone dextrose agar, and V8 agar. However, there was no conidia formation found in these conditions. Furthermore, the Raman spectroscopic fingerprinting databases for conidia of Bd and Dt are not currently available due to the technical difficulties.

**Fig 3:**
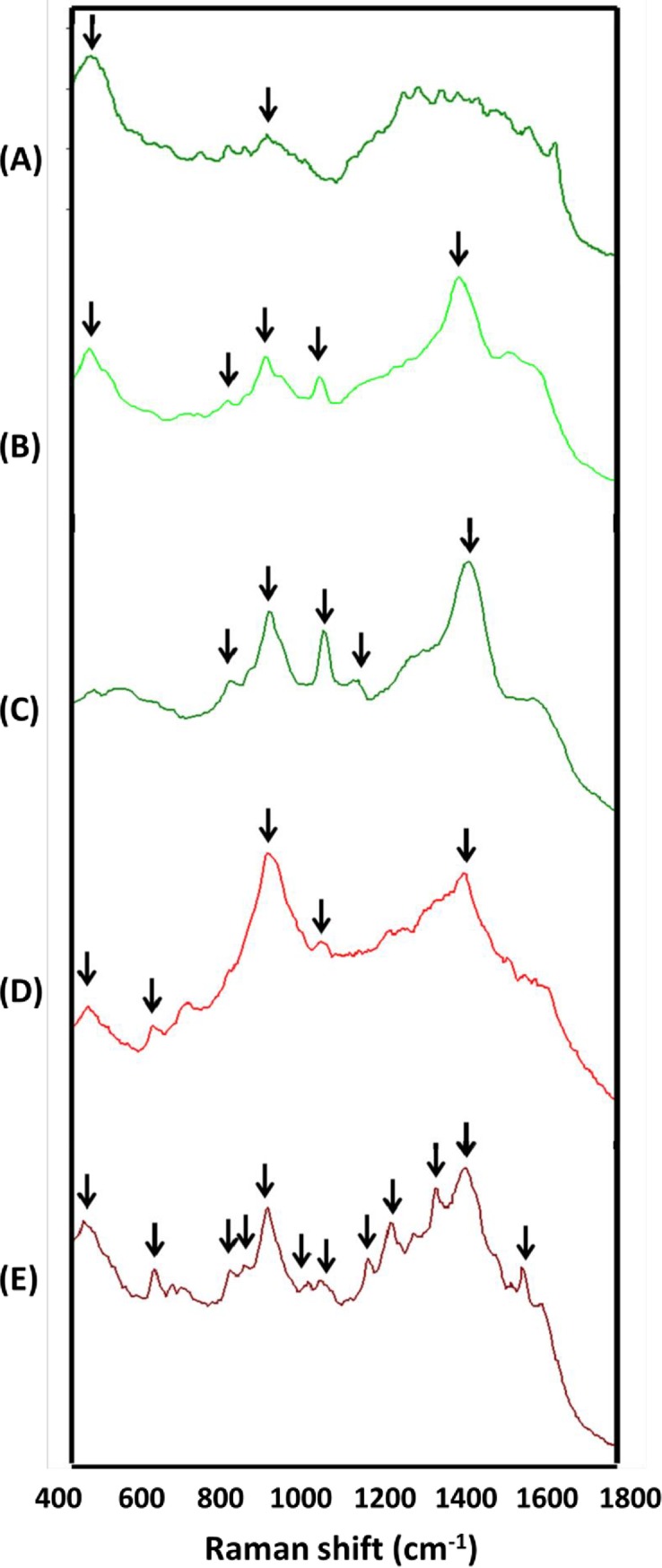
Raman fingerprints of banana samples with varying levels of symptoms collected from 12 different fields. The Raman spectra databases of banana samples including (A) healthy, (B) asymptomatic, (C) mildly symptomatic, (D) moderately symptomatic, and (E) severely symptomatic pseudostems were built. Average Raman spectra were obtained from five independent replications of the surface-enhanced Raman spectroscopy measurements.

A reliable method for the early monitoring of banana health is essential for formulating appropriate and timely disease management strategies to counteract the diseases affecting bananas. Traditional laboratory methods for diagnosing FWB involve lengthy assays including pathogen isolation, culturing, and morphological observation, as well as pathogenicity testing, which can take a few days to months [8]. One solution to this problem has been the use of advanced molecular techniques such as PCR [5, 6], multiplex PCR [7], real time-PCR [8, 9] LAMP [10], RealAmp [11, 12], and iiPCR [1] assays, all of which provide a more rapid diagnosis of FWB. However, use of these approaches for on-site diagnosis of FWB has been limited by their requirement of appropriate DNA extraction protocols, because it is sometimes difficult to detect Foc in infected banana pseudostem samples when the DNA quality from the tested samples is poor. In this study, therefore, we constructed the Raman spectroscopic fingerprints of various symptomatic banana pseudostem samples infected by Foc (Fig 3). The characteristic peaks of pseudostems with varying levels of symptoms were located at 447 cm^−1^ (for Foc-free pseudostems, Fig 3A), 447, 802, 896, 1044, and 1400 cm^−1^ (for asymptomatic pseudostems, Fig 3B); 802, 896, 1044, 1113, and 1400 cm^−1^ (for mildly symptomatic pseudostems, Fig 3C); 447, 614, 896, 1044 and 1400 cm^−1^ (for moderately symptomatic pseudostems, Fig 3D); and 447, 614, 802, 830, 896, 1039, 1044, 1163, 1212, 1332, 1400, and 1572 cm^−1^ (for severely symptomatic pseudostems, Fig 3E), respectively. In contrast to the characteristics of the samples with more severe symptoms, the Raman spectral features of asymptomatic and mildly symptomatic pseudostems were similar (but differentiable with those of Foc-free pseudostems). These data indicated that there seemed to be only slight changes in the overall molecular composition detected by Raman spectroscopy during the development of symptoms in pseudostems as they went from healthy to mildly symptomatic. In addition, the prominent SERS peaks at 896, 1044, and 1400 cm^-1^, which mainly come from fatty acid chains [34] and proline [38], could be consistently observed in both the asymptomatic and symptomatic pseudostems. There were more and a greater variation in the Raman spectra of the severely symptomatic pseudostems than in the other pseudostems, indicating that there seemed to be great change in the overall molecular composition detected by Raman spectroscopy when the pseudostems were severely infected by Foc. Moreover, the Raman peaks (e.g. at 447, 830, 1044, 1212, 1400, and 1572 cm^-1^) of the field banana samples did not always perfectly match the Raman peaks of the Foc mycelia, microconidia, and macroconidia because it is easy for Raman peaks (300–1800 cm^−1^) to be interfered with by the Raman signals from endogenous biomolecules [39].

Before this study, Raman spectroscopy had not been used for the diagnosis of banana diseases. In this study, the most reproducible characteristic Raman spectra (asymptomatic: 802, 896, and 1044 cm^−1^; mildly symptomatic: 896, 1044, and 1113 cm^−1^; moderately symptomatic: 614, 896, and 1044 cm^−1^; severely symptomatic: 830, 896, 1039, 1400, and 1572 cm^−1^; and 447 cm^−1^ as healthy control) were collected to differentiate field-infected bananas with varying levels of symptoms. A field detection evaluation was further performed to determine whether the spectroscopic method based on the reproducible characteristic Raman spectra was suitable for the diagnosis of FWB. For this purpose, we collected a total of 73 banana pseudostem samples (among them, there were 21 asymptomatic pseudostems and 52 symptomatic pseudostems, including 22 mildly symptomatic, 11 moderately symptomatic, and 19 severely symptomatic pseudostems) and 15 samples of banana infected with fungal pathogens other than Foc (3 samples for each disease) from 12 different fields that had been strongly affected by FWB for the Raman spectroscopic fingerprinting analysis and the three molecular detections. We chose three published methods for comparison, namely PCR-based pathogen-identification (MDIP), PCR-based in-planta detection (IPDP), and qPCR-based in-planta detection (IPDQP). As shown in Table 3, the detection rates of the Raman spectroscopic fingerprinting for the diagnosis of FWB in the asymptomatic, mildly symptomatic, moderately symptomatic, and severely symptomatic pseudostems were 16/21 (76.2%), 19/22 (86.4%), 9/11 (81.8%) and 17/19 (89.5%), respectively. As expected, all samples of banana infected with five fungal pathogens other than Foc yielded negative Foc detection results (including MDIP, IPDP, IPDQP, and SERS measurements) (Table 3). It is worth noting that the detection rates of the Raman spectroscopic fingerprinting, MDIP, IPDP, and IPDQP methods were 16/21 (76.20%), 7/21 (33.33%), 8/21 (38.10%), and 17/21 (81.0%) respectively, when the 21 asymptomatic pseudostems (each of the samples yielded at least two positive detection results within the four methods) were used as test samples. The detection rate for the asymptomatic samples when using the spectral method was 76.2%, which was comparable to that of the published molecular detection using real-time PCR (81.0%, IPDQP). In addition, after a 2-month incubation period, we were able recover Foc from those 21 asymptomatic pseudostems that progressed to being symptomatic by using the plate-out assay. It indicated that the field-detection results of this study were supported by those of traditional plate-out assays.

**Table 3:** Comparison of SERS measurement and the molecular detection methods for field detection.

Disease/Samples	Symptoms	No. positive/Total results (%)^d^^,^^e^			
		SERS^b^ measurements^c^	Molecular detection methods used in this study		
			MDIP^b^	IPDP^b^	IPDQP^b^
Fuearium wilt (FW)/Pseudostems (P)	No symptom^a^	16/21 (76.2%)	7/21 (33.3%)	8/21 (38.1%)	17/21 (81.0%)
FW/P	Mild^a^	19/22 (86.4%)	12/22 (54.5%)	18/22 (81.8%)	19/22 (86.4%)
FW/P	Moderate^a^	9/11 (81.8%)	9/11 (81.8%)	9/11 (81.8%)	10/11 (90.9%)
FW/P	Severe^a^	17/19 (89.5%)	11/19 (57.9%)	17/19 (89.5%)	18/19 (94.3%)
Cordana leaf spot/Leaves (L)	Pale yellowing	0/3 (0%)	0/3 (0%)	0/3 (0%)	0/3 (0%)
Deightoniella leaf spot/L	Black leaf streak	0/3 (0%)	0/3 (0%)	0/3 (0%)	0/3 (0%)
Alternaria speckle/L	Black spot	0/3 (0%)	0/3 (0%)	0/3 (0%)	0/3 (0%)
Anthracnose/L	Necrosis	0/3 (0%)	0/3 (0%)	0/3 (0%)	0/3 (0%)
Botryosphaeria crown rot/Fruits	Crown rot	0/3 (0%)	0/3 (0%)	0/3 (0%)	0/3 (0%)

^a^Mild symptoms = less than 1/3 area of pseudostem necrosis; moderate symptoms = less than 2/3 but equal to or more than 1/3 area of pseudostem necrosis; severe symptoms = equal to or more than 2/3 area of pseudostem necrosis

^b^SERS: surface-enhanced Raman spectroscopy; MDIP: molecular detection of isolated pathogen; IPDP: *in-planta* detection with PCR; IPDQP: in-planta detection with real-time PCR

^c^positive SERS measurements mean the symptomatic/asymptomatic sample yielded the specific patterns of the most reproducible characteristic Raman spectra

^d^A total of 73 banana pseudostems with varying levels of symptoms (among them, 21 asymptomatic and 52 symptomatic pseudostems, including 22 mildly symptomatic, 11 moderately symptomatic, and 19 severely symptomatic pseudostems) were used for field detection

^e^A total of 15 field samples of banana infected with fungal pathogens other than *F*. *oxysporum* f. sp. *cubense* (3 samples for each disease) were used for field detection

The Raman spectroscopic fingerprinting assay is a simple and rapid method (requiring no DNA extraction procedures) for differentiating conidia or mycelia of Foc from those of other fungal pathogens collected from bananas, such as Colm, Dt, Aa, Bd, and Corm, as well as for the diagnosis of FWB on pseudostem samples with varying levels of symptoms. An acceptable field-detection rate for FWB detection by the spectroscopic fingerprinting assay was observed among the results generated from the 73 random field-collected Foc-infected pseudostems with varying levels of symptoms, including asymptomatic pseudostems. In conclusion, to our knowledge, this is the first study to report the Raman spectroscopic fingerprinting databases of phytopathogenic fungi. The Raman spectroscopic fingerprinting assays developed in this study provide an alternative to conventional PCR and real-time PCR assays for the field-detection of FWB. The spectroscopic fingerprinting assays have the potential to serve as a rapid and simple tool for the routine diagnosis of FWB.

## Supporting information

S1 Fig Raman fingerprints of banana samples infected with fungal pathogens other than *F*. *oxysporum* f. sp. *cubense*. The Raman spectra databases of banana samples with (A) pale yellowing, (B) black leaf streak, (C) black spot, and (D) necrosis on leaves, and (E) crown rot on fruit were built. Average Raman spectra were obtained from three independent replications of the surface-enhanced Raman spectroscopy measurements.
(TIF)

## References

[1] Lin YH, Lin YJ, Chang TD, Hong LL, Chen TY, and Chang PFL. Development of a TaqMan probe-based insulated isothermal polymerase chain reaction (iiPCR) assay for detection of *Fusarium oxysporum* f. sp. *cubense* race 4. PLoS ONE. 2016;11: e0159681 10.1371/journal.pone.0159681 27448242 PMC4957775

[2] Smith SN. An overview of ecological and habitat aspects in the genus Fusarium with special emphasis on the soil-borne pathogenic forms. Plant Pathol Bull. 2007;16: 97–120.

[3] Warman NM, Aitken EAB. The movement of *Fusarium oxysporum* f. sp. *cubense* (sub-tropical race 4) in susceptible cultivars of banana. Front Plant Sci. 2018;9: 1748 10.3389/fpls.2018.01748 30538716 PMC6277567

[4] Schaad NW, Frederick RD, Shaw J, Schneider WL, Hickson R, Petrillo MD, et al Advances in molecular-based diagnostics in meeting crop biosecurity and phytosanitary issues. Annu Rev Phytopathol. 2003;41: 305–324. 10.1146/annurev.phyto.41.052002.095435 14527331

[5] Lin YH, Chang JY, Liu ET, Chao CP, Huang JW, and Chang PFL. Development of a molecular marker for specific detection of *Fusarium oxysporum* f. sp. *cubense* race 4. Eur J Plant Pathol. 2009;123: 353–365.

[6] Fraser-Smith S, Czislowski E, Meldrum RA, Zander M, O’Neill W, Balali GR, et al Sequence variation in the putative effector gene SIX8 facilitates molecular differentiation of *Fusarium oxysporum* f. sp. *cubense*. Plant Pathol. 2014;63: 1044–1052.

[7] Dita MA, Waalwijk C, Buddenhagen IW, Souza MT Jr, and Kema GHJ. A molecular diagnostic for tropical race 4 of the banana fusarium wilt pathogen. Plant Pathol. 2010;59: 348–357.

[8] Lin YH, Su CC, Chao CP, Chen CY, Chang CJ, Huang JW, et al A molecular diagnosis method using real-time PCR for quantification and detection of *Fusarium oxysporum* f. sp. *cubense* race 4. Eur J Plant Pathol. 2013;135: 395–405.

[9] Yang LL, Sun LX, Ruan XL, Qiu DY, Chen DH, Cai XQ, et al Development of a single-tube duplex real-time fluorescence method for the rapid quantitative detection of *Fusarium oxysporum* f. sp. *cubense* race 1 (FOC1) and race 4 (FOC4) using TaqMan probes. Crop Prot. 2015;68: 27–35.

[10] Li B, Du J, Lan C, Liu P, Weng Q, and Chen Q. Development of a loop-mediated isothermal amplification assay for rapid and sensitive detection of *Fusarium oxysporum* f. sp. *cubense* race 4. Eur J Plant Pathol. 2013;135: 903–911.

[11] Peng J, Zhang H, Chen F, Zhang X, Xie Y, Hou X, et al Rapid and quantitative detection of *Fusarium oxysporum* f. sp. *cubense* race 4 in soil by real-time fluorescence loop-mediated isothermal amplification. J Appl Microbiol. 2014;117: 1740–1749. 10.1111/jam.12645 25200557

[12] Zhang X, Zhang H, Pu J, Qi Y, Yu Q, Xie Y, et al Development of a real-time fluorescence loop-mediated isothermal amplification assay for rapid and quantitative detection of *Fusarium* oxysporum f. sp. *cubense* tropical race 4 in soil. PLoS ONE. 2013;8: e82841 10.1371/journal.pone.0082841 24376590 PMC3869718

[13] Zhao X, Li M, and Xu Z. Detection of foodborne pathogens by surface enhanced raman spectroscopy. Front Microbiol. 2018;9: 2857 10.3389/fmicb.2018.02857 29946307 PMC6005832

[14] Wu X, Han C, Chen J, Huang YW, and Zhao Y. Rapid detection of pathogenic bacteria from fresh produce by filtration and surface-enhanced Raman spectroscopy. JOM 2016;68: 1156–1162.

[15] Liu Y, Zhou H, Hu Z, Yu G, Yang D, and Zhao J. Label and label-free based surface-enhanced Raman scattering for pathogen bacteria detection: a review. Biosens Bioelectron. 2017;94: 131–140. 10.1016/j.bios.2017.02.032 28262610

[16] Pallaoro A, Hoonejani MR, Braun GB, Meinhart CD, and Moskovits M. Rapid identification by surface-enhanced Raman spectroscopy of cancer cells at low concentrations flowing in a microfluidic channel. Acs Nano. 2015;9: 4328–4336. 10.1021/acsnano.5b00750 25781324

[17] Kong K, Kendall C, Stone N, and Notingher I. Raman spectroscopy for medical diagnostics—From *in-vitro* biofluid assays to *in*-*vivo* cancer detection. Adv Drug Delivery Rev. 2015;89: 121–134. 10.1016/j.addr.2015.03.009 25809988

[18] Pang S, Yang T, and He L. Review of surface enhanced Raman spectroscopic (SERS) detection of synthetic chemical pesticides. TrAC Trends in Anal Chem. 2016;85: 73–82.

[19] Jiang Y, Sun DW, Pu H, and Wei Q. Surface enhanced Raman spectroscopy (SERS): A novel reliable technique for rapid detection of common harmful chemical residues. Trends Food Sci Technol. 2018;75: 10–22.

[20] Rajapandiyan P, Tang WL, and Yang J. Rapid detection of melamine in milk liquid and powder by surface-enhanced Raman scattering substrate array. Food Control. 2015;56: 150–155.

[21] Hu Y, Feng S, Gao F, Li-Chan EC, Grant E, and Lu X. Detection of melamine in milk using molecularly imprinted polymers–surface enhanced Raman spectroscopy. Food Chem. 2015;176: 123–129. 10.1016/j.foodchem.2014.12.051 25624214

[22] Brauchle E, and Schenke-Layland K. Raman spectroscopy in biomedicine˗non-invasive *in vitro* analysis of cells and extracellular matrix components in tissues. Biotechnol J. 2013;8: 288–297. 10.1002/biot.201200163 23161832 PMC3644878

[23] Craig AP, Franca AS, and Irudayaraj J. Surface-enhanced Raman spectroscopy applied to food safety. Annu Rev Food Sci Technol. 2013;4: 369–380. 10.1146/annurev-food-022811-101227 23297774

[24] Feng S, Lin J, Huang Z, Chen G, Chen W, Wang Y, et al Esophageal cancer detection based on tissue surface-enhanced Raman spectroscopy and multivariate analysis. Appl Phys Lett. 2013;102: 043702.

[25] Tu Q, and Chang C. Diagnostic applications of Raman spectroscopy. Nanomedicine. 2012;8: 545–558. 10.1016/j.nano.2011.09.013 22024196

[26] Stiles PL, Dieringer JA, Shah NC, and Van Duyne RP. Surface-enhanced Raman spectroscopy. Annu Rev Anal Chem. 2008;1: 601–626. 10.1146/annurev.anchem.1.031207.112814 20636091

[27] Vendrell M, Maiti KK, Dhaliwal K, and Chang YT. Surface-enhanced Raman scattering in cancer detection and imaging. Trends Biotechnol. 2013;31: 249–257. 10.1016/j.tibtech.2013.01.013 23416096

[28] Moore T, Moody A, Payne T, Sarabia G, Daniel A, and Sharma B. *In vitro* and *in vivo* SERS biosensing for disease diagnosis. Biosensors. 2018;8: 46. 10.3390/bios8020046 PMC6022968 29751641

[29] Pînzaru SC, Falamaş A, Dehelean C, Morari C, and Venter M. Double amino functionalized Ag nanoparticles as SERS tags in Raman diagnostic. Croatica Chemica Acta. 2013;86: 233–244.

[30] Feng S, Huang S, Lin D, Chen G, Xu Y, Li Y, et al Surface-enhanced Raman spectroscopy of saliva proteins for the noninvasive differentiation of benign and malignant breast tumors. Int J Nanomedicine. 2015;10: 537–547. 10.2147/IJN.S71811 25609959 PMC4298339

[31] Nash SM, Snyder WC. Quantitative estimations by plate counts of propagules of the bean root rot Fusarium in field soils. Phytopathology. 1962;52: 567–572.

[32] Xie Y, Xu L, Wang Y, Shao J, Wang L, Wang H, et al Label-free detection of the foodborne pathogens of Enterobacteriaceae by surface-enhanced Raman spectroscopy. Analytical Methods. 2013;5: 946–952.

[33] Jarvis RM, Brooker A, and Goodacre R. Surface-enhanced Raman spectroscopy for bacterial discrimination utilizing a scanning electron microscope with a Raman spectroscopy interface. Anal Chem. 2004;76: 5198–5202. 10.1021/ac049663f 15373461

[34] Ellis DI, Cowcher DP, Ashton L, O'Hagan S, and Goodacre R. Illuminating disease and enlightening biomedicine: Raman spectroscopy as a diagnostic tool. Analyst. 2013;138: 3871–3884. 10.1039/c3an00698k 23722248

[35] Mahadevan-Jansen A, and Richarda-Kortum R. Raman spectroscopy for cancer detection: a review. Conf Proc IEEE Eng Med Biol Soc. 1997;6: 2722–2728.

[36] Barr H, Dix T, and Stone N. Optical spectroscopy for the early diagnosis of gastrointestinal malignancy. Lasers Med Sci. 1998;13: 3–13.

[37] Berg RW, Shim I, White PC, and Abdali S. Raman optical activity and Raman spectra of amphetamine species-quantum chemical model calculations and experiments. Am J Anal Chem. 2012;3: 410–421.

[38] Frank CJ, McCreery RL, and Redd DC. Raman spectroscopy of normal and diseased human breast tissues. Anal Chem.1995;67: 777–783. 10.1021/ac00101a001 7762814

[39] Lin X, Wang Y, Wang L, Lu Y, Li J, Lu D, et al Interference-free and high precision biosensor based on surface enhanced Raman spectroscopy integrated with surface molecularly imprinted polymer technology for tumor biomarker detection in human blood. Biosens Bioelectron. 2019;143: 111599 10.1016/j.bios.2019.111599 31476600

[40] Wetzel H, Gerischer H, and Pettinger B. Surface enhanced raman scattering from silver-halide and silver-pyridine vibrations and the role of silver ad-atoms. Chem Phys Lett. 1981;78: 392–397.

[41] De-Gussem K, Vandenabeele P, Verbeken A, and Moens L. Raman spectroscopic study of Lactarius spores (Russulales, Fungi). Spectrochim Acta A Mol Biomol Spectrosc. 2005;61: 2896–2908. 10.1016/j.saa.2004.10.038 16165029

[42] De-Veij M, Vandenabeele P, De-Beer T, Remon JP, and Moens L. Reference database of Raman spectra of pharmaceutical excipients. J Raman Spectrosc. 2009;40: 297–307.

[43] Mathlouthi M, Seuvre AM, and Koenig L. J. F.t.-i.r. and laser-Raman spectra of thymine and thymidine. Carbohydr Res. 1984;134: 23–38.

[44] Fan Y, Lai K, Rasco BA, and Huang Y. Analyses of phosmet residues in apples with surface-enhanced Raman spectroscopy. Food Control. 2014;37: 153–157.

[45] Otto C, van den Twee TJJ, de Mu1 FFM, and Greve J. Surface-enhanced Raman spectroscopy of DNA bases. J Raman Spectrosc. 1986;17: 289–298.

