# Supplementary figures and images for: Construction of Raman spectroscopic fingerprints for the detection of Fusarium wilt of banana in Taiwan
Source: PLoS One. 2020 Mar 16;15(3):e0230330. doi: 10.1371/journal.pone.0230330 (PMC7075571; doi:10.1371/journal.pone.0230330)

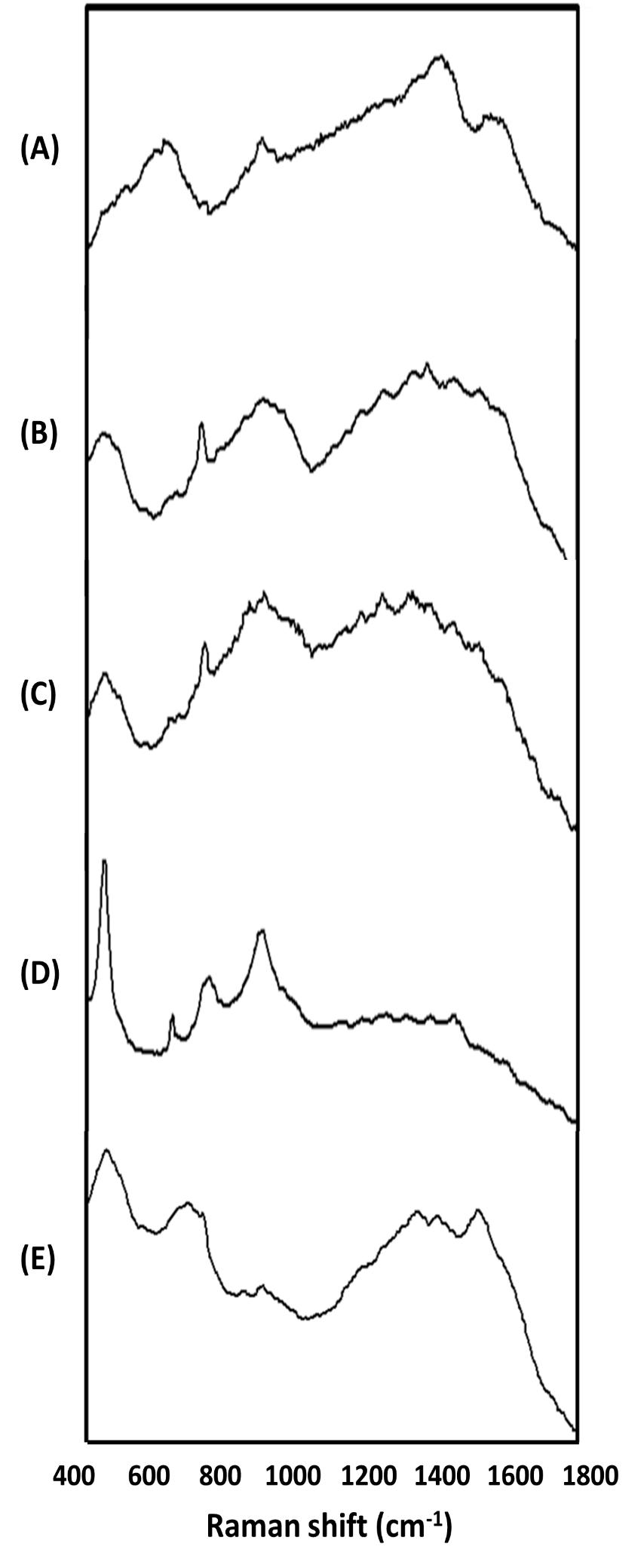

Supplement: S1 Fig — The Raman spectra databases of banana samples with (A) pale yellowing, (B) black leaf streak, (C) black spot, and (D) necrosis on leaves, and (E) crown rot on fruit were built. Average Raman spectra were obtained from three independent replications of the surface-enhanced Raman spectroscopy measurements. (TIF) [file pone.0230330.s001.tif]
